# Insulin resistance induced by pesticides is overcome by pancreatic islet adaptation in a mouse model of Parkinson's disease

**DOI:** 10.1016/j.molmet.2026.102388

**Published:** 2026-05-26

**Authors:** Ambrine Arrar, Nour Mesto, Roxane Descaillot, Muris Humo, Aude De Cesar, Damien Gaillard, Latifa Lakhdar, Johann Vulin, Claire Aufauvre, Raphaëlle Baumier, Camille Bellières, Jacques Imbert, Prunelle Perrier, Emilien L. Jamin, Romain Vuillaume, Jean-Noël Arsac, Anne Fougerat, Magalie A. Ravier, Thierry Baron, Laurence Gamet-Payrastre, Safia Costes

**Affiliations:** 1 IGF, Univ Montpellier, CNRS, INSERM, Montpellier, France; 2 ANSES, University of Lyon, Lyon, France; 3 MGX-Montpellier GenomiX, Univ Montpellier, CNRS, INSERM, Montpellier, France; 4 Toxalim (Research Center in Food Toxicology), INRAE, ENVT, INP-Purpan, UPS, Toulouse University, Toulouse, France; 5 MetaboHUB-Metatoul, National Infrastructure of Metabolomics and Fluxomics, Metatoul-AXIOM, Toulouse, France

**Keywords:** Pesticides, Insulin resistance, Pancreatic beta-cell, Parkinson's disease

## Abstract

Exposure to certain pesticides appears to be involved in type 2 diabetes and Parkinson's disease onset which are reported to be epidemiologically associated. While the exact causes of this association and the deleterious mechanisms linking these aging-associated diseases are not fully known, it seems important to assess the extent to which environmental factors such as pesticides could be involved. Our aim was to evaluate the consequences of chronic dietary exposure to a mixture of pesticides at levels below the Acceptable Daily Intake in transgenic mice predisposed to develop motor disorders. Male mice expressing mutated A53T human α-synuclein (M83) and wild-type mice were fed either a control or a diet enriched with 6 pesticides (captan, boscalid, chlorpyrifos, thiachloprid, thiofanate, ziram) for 50 weeks. Exposure to pesticides led to body weight gain and insulin resistance in wild-type and M83 mice, caused, at least in part, by a reduction in insulin receptor levels in liver, skeletal muscle and adipose tissue. However, only M83 mice exposed to pesticides showed early motor deficits associated with decreased insulin receptor levels in midbrain and striatum. While pesticides promoted glucose intolerance in wild-type mice, M83 mice surprisingly showed improved glucose tolerance accompanied by a significant increase in pancreatic beta-cell mass and function. Transcriptomic analysis further revealed an enrichment of genes involved in amino-acid metabolism in M83 mouse islets with abundant α-synuclein expression. Overall, exposure to pesticides led to insulin resistance, which can be overcome thanks to a previously unrecognized role of α-synuclein in beta-cell adaptation.

## Highlights

• Pesticide mix induces body weight gain and insulin resistance in mice.
• Pesticide mix leads to decreased insulin receptor in insulin target tissues.
• Wild-type mice exposed to pesticides are glucose intolerant.
• Parkinson's disease mice exposed to pesticides show improved glucose tolerance.
• High α-synuclein promotes islet adaptation to pesticide-induced insulin resistance.

## Graphical abstract

**Figure:**
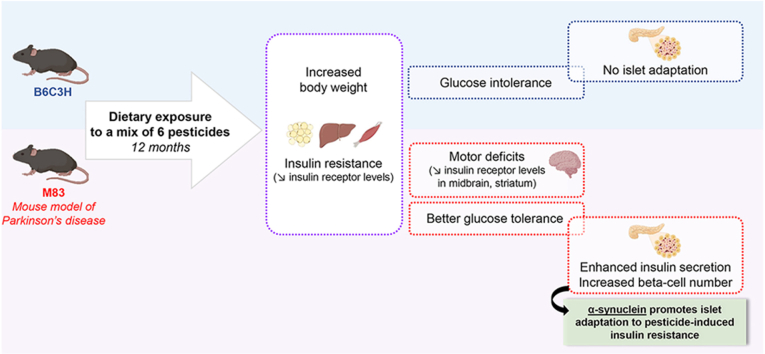

## 1 Introduction

Epidemiological evidences provide proof of concept that certain pesticides are involved in metabolic disorders [[1], [2], [3], [4]], but also in the pathophysiology of Parkinson's disease (PD) [[5], [6], [7]]. In addition, large prospective cohort studies reported that type 2 diabetes (T2D) and PD are epidemiologically associated [[8], [9], [10]], including an elevated risk of developing PD in patients with T2D [8,9].

T2D and PD are both irreversible aging-associated diseases that are turning into epidemics worldwide. They belong to the group of protein misfolding diseases, which share aggregation of misfolded/amyloid proteins in long-lived cells [11]. PD, which belongs to a family of disorders termed synucleinopathies, is the second most common neurodegenerative disease in the world and is a major cause of disability in the elderly. PD is characterized by degeneration of dopaminergic neurons in the *substantia nigra* and their extensions in the striatum, a brain area essential for motor control. Its characteristic lesions, Lewy bodies and neurites, contain α-synuclein under an aggregated and serine 129 phosphorylated (^Ser129^P) form [12]. T2D is a metabolic disorder characterized by chronic hyperglycemia and recognized as a major public health issue. Reduction in insulin sensitivity (in liver, muscle, adipose tissue), most often attributed to high-caloric food intake and consequent obesity, is a well-known risk factor for T2D. However, most obese individuals do not develop diabetes because they are able to compensate for insulin resistance by an adaptive rise in insulin secretion by pancreatic beta-cells [13]. T2D occurs when beta-cells are exhausted and fail to adequately increase insulin secretion to meet demands to counteract insulin resistance, and this failure may be exacerbated by a reduction in beta-cell mass over time [14]. Further highlighting the closely shared molecular pathology between T2D and PD, the islet in T2D is also characterized by amyloid deposits derived from Islet Amyloid Polypeptide (IAPP), the major aggregating component in human T2D [15,16]. In addition, α-synuclein, whose amyloidogenic properties are central to the development of PD, is expressed in pancreatic beta-cells [17,18], and its accumulation and aggregation were observed in subjects with T2D and/or PD [19,20]. However, the roles of α-synuclein on beta-cell function and integrity remain to be clarified [17,18]. Beyond these shared amyloidogenic properties, there is also clear evidence that insulin signalling is involved in the pathogenesis of PD [21], therefore pointing to - peripheral and central - insulin resistance as a common trigger for both PD and T2D.

Therefore, some common initiating processes (insulin resistance/protein aggregation) suggest a common genetic predisposition and/or the existence of common pathogenic mechanisms for these two pathologies, but it remains to understand what are the deleterious molecular mechanisms linking T2D and PD, and to assess to what extent environmental factors such as pesticides could be involved in these alterations.

Whereas occupational exposure to some pesticides is now considered a risk factor for PD [[5], [6], [7]] and T2D [1,3,4], very few studies have addressed the question of dietary exposure to a pesticide mixture at nontoxic doses for the general population. Indeed, consumers are mainly exposed through food intake to mixtures of pesticides at low doses throughout their lives, and it is difficult to assess the consequences of such chronic exposure on health, based on the individual effect of each compound. In this study, we selected six pesticides (insecticides/fungicides: boscalid, captan, chlorpyrifos, thiacloprid, thiofanate, and ziram) from among those used to treat apple orchards in southern France, a mixture of which is commonly used in fruit and vegetable cultivation within the EU [2]. To best mimic exposure of the general population, we exposed mice, via their diet, to a mixture of these 6 pesticides at the Acceptable Daily Intake (ADI) for each compound (i.e. an estimate of the amount that can be ingested on a daily basis over a lifetime without appreciable risk to human health). We previously clearly reported metabolic and hepatic alterations caused by this chronic dietary exposure in C57Bl/6J mice [2], but peripheral and central insulin resistance, pancreatic and neuronal integrity were not investigated.

In the present study, we assessed the consequences of the above-mentioned environmental exposure in a transgenic mouse model of α−synucleinopathy and PD (M83 mice) [22] overexpressing the A53T mutated human α-synuclein involved in some genetic PD cases. Whereas homozygous M83 mice develop characteristic motor symptoms (reduced ambulation, balance disorders, paralysis) between 8 and 16 months of life [22], hemizygous mice may not display any clinical signs beyond the age of 22 months. In addition, given that expression of A53T mutated human α-synuclein is governed by the mouse prion protein also found in endocrine pancreas [23,24], this mouse model may also provide an opportunity to investigate its role in pancreatic islets. Therefore, using M83 hemizygous mice prone to develop motor deficits very late, we aimed to: i) Evaluate the metabolic and neuronal consequences of chronic (12 months) dietary exposure to the cocktail of pesticides, ii) Highlight the existence of alteration/compensation mechanisms common to pancreas and brain under such environmental exposure.

## 2 Material and methods

### 2.1 Chemicals

High-purity pesticides were purchased from Sigma–Aldrich. Information on pesticides’ toxicity was obtained from the E-Phy database (https://ephy.anses.fr/).

### 2.2 Pesticide chow

Chemical families and functions of pesticides (ziram, thiofanate, captan, chlorpyrifos, boscalid, thiachloprid) are described in Table S1. Exposure of mice at the ADI of each pesticide is defined in mg/kg BW (Body Weight) per day as described [2].

The quantities of pesticides incorporated in the diet have already been reported [2] and are described in Table S1. The standard synthetic diet from Animal Feed Preparation Unit (SAAJ, INRAE) contains 63% carbohydrate, 5% fat, 22% protein, 2% cellulose, 1% vitamins [vitaminic mixture 200, Scientific Animal Food Engineering (SAFE)] and 7% minerals (205b, SAFE France). Control and pesticides enriched feed were prepared as described [2]. The final concentrations of pesticides present in the diet were quantifed by Eurofins (Table S1). Control feed analysis confirmed that none of the pesticides studied were detected.

### 2.3 Animal experiment

Animal experiments were conducted at ANSES facility following the EU guidelines for laboratory animal use and care (Agreement D693870801), and was approved by the ethic committee (ComEth ANSES/ENVA/UPEC) and by the French Ministry of Higher Education, Research and Innovation (Authorization n°22–52). The animals were housed in Sealsafe Plus GM500 ventilated cages (Tecniplast).

M83 transgenic mice (B6; C3H-Tg[SNCA]83Vle/J) express A53T mutated human α-synuclein protein under the control of the prion protein promoter [22]. In this study, only M83 hemizygous mice were used. B6C3H mice served as wild-type strain for comparison with M83 mice on the same genetic background. Both strains were treated similarly and experiments performed concurrently to allow direct comparison of the results. After 3 weeks on standard chow diet, all fifteen-week-old male mice (at week 0) were exposed to either a pesticide-enriched or pesticide-free diet, which was continued for 50 weeks to mimic a ∼30-year exposure in humans [25]. Mice were randomly housed four or five per cage in a temperature-controlled room on a 12h light/dark cycle, and received water and food *ad libitum*. Four groups of 35 animals each were followed in this study: B6C3H males non-exposed, B6C3H males exposed, M83 males non-exposed, M83 males exposed. Mice were randomly distributed into groups. During 50 weeks of exposure, BW and food intake were monitored weekly; blood glucose was measured at the tail vein using Freestyle Optium Neo glucometer (Abbott). 26 and 50 weeks postexposure, 6 to 20 mice from each experimental group were randomly sacrificed by cervical dissociation. Blood was collected into EDTA-K3 tubes (Labellians) for plasma preparation by centrifugation (6000 rpm, 10 min, 4 °C). Pancreases were collected, weighed and either fixed for histological analysis or collagenase-digested for islet isolation. Livers, soleus muscles, subcutaneous white adipose tissue, midbrains (containing the *substantia nigra pars compacta*) and striatum were collected, frozen in liquid nitrogen, and conserved at −80 °C for further protein expression analyses.

### 2.4 Open–field tests and dopaminergic neuron count

The open-field test was conducted during the light phase to assess locomotor activity and identify potential signs of hypokinesia (reduced movement) and bradykinesia (slowed movement execution) in B6C3H and M83 mice. Each group consisted of 15 mice, with four experimental groups: B6C3H non-exposed, B6C3H exposed, M83 non-exposed and M83 exposed. Mice were placed in one corner of a 40 × 40 × 40 cm opaque chamber with an open top and allowed to explore freely for 5 min [26]. The test was performed at five time points: baseline (prior to pesticide exposure), and at 12, 24, 36, and 40 weeks after the initiation of pesticide exposure. Locomotor activity was tracked using ANY-maze tracking software (Version 7.0, Stoelting Co.), which recorded the mice's body positions throughout the session. The total distance traveled and the average speed during the exploration period were analyzed to evaluate spontaneous motor activity and locomotor velocity. Procedure for counting dopaminergic neurons is detailed in Supplemental Methods.

### 2.5 Insulin tolerance test

Experiments were performed on conscious mice at weeks 16, 36 and 48 following pesticide exposure. Mice were fasted for 6 h and received an intraperitoneal insulin load (0.75 U/kg BW) (Umuline rapide, Lilly). Blood glucose was measured at the tail vein using Freestyle Optium Neo glucometer before (0 min) and 15, 30, 60, 90, 120 min after the insulin load.

### 2.6 Glucose tolerance test

Experiments were conducted on conscious mice. For the intraperitoneal glucose tolerance test at week 16 or the oral glucose tolerance tests at weeks 36 and 48, mice were fasted for 6 h and received a glucose load (1.5 g/kg BW). Glycemia was measured at the tail vein with a Freestyle Optium Neo glucometer before (0 min) and 10, 30, 60, 90, 120, 150 min after the glucose load. Blood was collected from the submandibular vein using a lancet into EDTA-K3 tubes before the glucose load (Basal) and 15 min after (T15). Plasma was stored at −80 °C until insulin assay.

### 2.7 Plasma insulin and glucagon assays

Plasma insulin and plasma glucagon were assayed using ultrasensitive mouse insulin ELISA or mouse glucagon ELISA kit (Crystal Chem), respectively.

### 2.8 Histological analysis and immunostaining of pancreases

Pancreases were processed, stained with hematoxylin and eosin (H&E) and scanned as reported [27]. Islet number and area were quantified with NDP.view software, version 2.9.29 (Hamamatsu). For immunohistochemistry, deparaffinised sections were treated as described [27]. Sections were incubated at 4 °C overnight with primary antibodies (Table S2), then with secondary antibodies (Table S2) and DAPI during 1 h at room temperature. Sections were mounted using Mowiol (Sigma–Aldrich) and imaged using an AxioImager Apotome microscope (Zeiss) using the ZenBlue software. Analyses were then performed manually using ImageJ (Fiji): measurement of beta-cell area per islet, number of beta-cells per islet, percentage of insulin-positive cells [27]. On average, 70 islets/section were analyzed. Experimenters were blinded to group assignment.

### 2.9 Western blot analysis

Mouse islets, livers, muscles or adipose tissue were lysed in NP40 lysis buffer [28]. Midbrain and striatum homogenates were mixed with TD4215 denaturing buffer (final concentration: 4% SDS, 2% β mercaptoethanol, 192 mM glycine, 25 mM Tris, 5% sucrose). All tissue samples were then sonicated and centrifuged at 10,000 rpm at 4 °C for 10 min. Western blotting was conducted as described [28,29]. Membranes were incubated overnight at 4 °C with primary antibodies (Table S2) and 1 h with secondary antibodies (Table S2). Proteins were visualized by chemiluminescence on a ChemiDoc Camera (Biorad), and the Image J software was used to quantify protein expression levels [28,29].

### 2.10 Mouse islet isolation and gene expression

Pancreatic islets were isolated after collagenase digestion of pancreases [27] obtained from B6C3H and M83 male mice fed control chow for 50 weeks. Extended protocole on gene expression studies can be found in Supplemental Methods.

### 2.11 Statistical analysis

Unless otherwise indicated, data are mean ± SEM for the indicated number of observations. GraphPad Prism 9.2.0 software was used. Statistically significant differences between groups were evaluated by Student's two-tailed unpaired or paired t test (pairwise comparisons), or by one-way or two-way ANOVA (multiple comparisons). For ANOVA, when the global factor effect was significant, post-hoc tests were performed to get adjusted p-values. We used Sidak's post-hoc tests for comparison between selected pairs of columns. A p-value less than 0.05 was considered significant.

## 3 Results

### 3.1 Increased body weight in mice exposed to pesticides compared to those fed control chow

**Figure 1:**
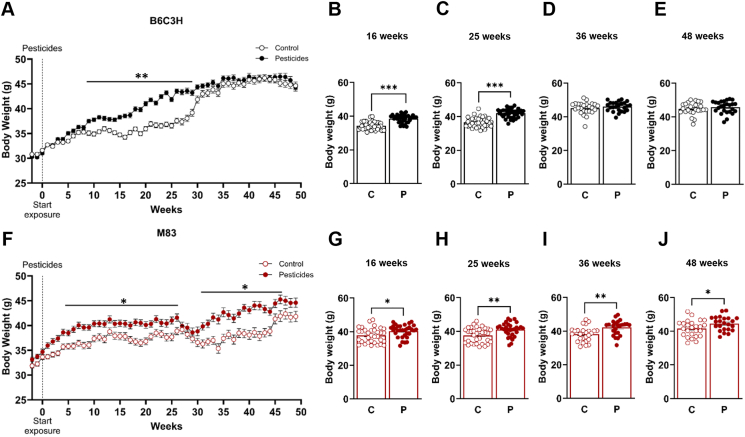
**Body weight of B6C3H and M83 mice exposed or not to pesticides.** (**A, F**) Body weight (g) of B6C3H (in black) and M83 (in red) mice exposed or not to pesticides. The dotted lines delimit the body weight two weeks prior and after feeding the mice control chow (empty circles, C) or pesticide chow (filled circles, P). Mice (n = 35 mice per group at start) were exposed for 50 weeks. The bar graphs show body weight at 16 weeks (**B, G**), 25 weeks (**C, H**), 36 weeks (**D, I**) and 48 weeks (**E, J**). Data are presented as mean ± SEM. ∗p < 0.05; ∗∗p < 0.01; ∗∗∗p < 0.001 compared to mice fed control chow as determined by two-way ANOVA (**A, F**) and Student's *t* test (**B, C, D, E, G, H, I, J**).

Pesticide levels assessed in pellet before the beginning of the exposure confirmed their presence in the diet at levels slightly below the expected quantities (Table S1). As described [2], actual pesticide levels to which mice were exposed was calculated after accounting for animal body weight and food intake for the duration of exposure, and these levels were below the ADI for each pesticide (Fig. S1). After assessement of pesticide exposure, we compared the body weight of the 4 groups of mice. Between 9 and 29 weeks postexposure, wild-type (B6C3H) mice fed the pesticide mixture displayed a significant increase in body weight in comparison to those fed control chow (Figure 1A–C). Indeed, 25 weeks postexposure, the body weight gain in pesticide-exposed B6C3H mice was increased by 2-fold in comparison to mice fed control chow (10.87 ± 2.16 g vs. 4.95 ± 2.05 g, respectively). However, after 30 weeks, B6C3H mice no longer exhibited changes in body weight upon pesticide exposure (Figure 1A,D, E). M83 mice fed the pesticide mixture also gained more weight than mice fed control chow (Fig. 1F). In contrast to B6C3H, this increase in body weight was notable throughout the entire exposure period from 5 weeks to the end (Figure 1F–J). 25 weeks postexposure, the body weight gain in pesticide-exposed M83 mice was increased by 1.5-fold in comparison to mice fed control chow (6.14 ± 3.88 g vs. 4.09 ± 3.84 g, respectively). Food intake was comparable in mice fed pesticide chow compared to mice fed control chow for both B6C3H and M83 (Fig. S2) and could thus not account for differences in body weight gain. Overall, our data indicate that chronic dietary exposure of both B6C3H and M83 mice to a mixture of pesticides at levels below the ADI induced a greater body weight gain than in mice fed control chow, with a persisting effect in M83 mice.

### 3.2 Reduced locomotor activity in M83 mice exposed to pesticide chow

**Figure 2:**
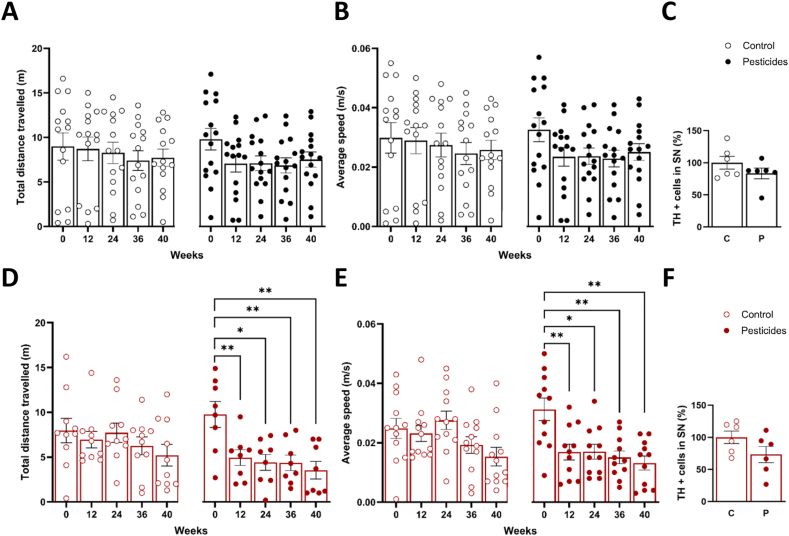
**Locomotor activity of B6C3H and M83 mice exposed or not to pesticides.** Total distance travelled (in meter) for B6C3H (**A**, black) and M83 (**D**, red) mice exposed for the indicated weeks to the pesticide mixture. Average speed (in meter per second) for B6C3H (**B**) and M83 (**E**) mice exposed for the indicated weeks to the pesticide mixture. Number of tyrosine hydroxylase positive cells in the substantia nigra (SN) of B6C3H (**C**, black) and M83 (**F**, red) mice exposed (P) or not (C) to pesticides. n = 6–15 mice/group. Results are presented as mean ± SEM; ∗p < 0.05, ∗∗p < 0.01 as determined by analysis of variance (ANOVA) followed by Dunnett's post-hoc tests for paired comparisons and by Student's *t* test.

The M83 mouse being a model of α-synucleinopathy and PD, spontaneous locomotor activity was assessed. A significant reduction in commonly used motor parameters including total distance travelled and average speed was observed only in M83 mice exposed to pesticides (Figure 2A,B, D, E). Whereas this effect became evident as early as 12 weeks after exposure and persisted throughout the entire exposure period, M83 mice exposed to pesticides showed no significant changes in the number of dopaminergic neurons (TH+) (Figure 2C,F). In addition, since B6C3H have gained similar weight than M83 mice under pesticide exposure, the locomotor deficits observed solely in M83 mice is unlikely to be caused by this weight gain.

### 3.3 Insulin resistance and glucose intolerance in B6C3H mice exposed to pesticide chow compared to mice fed control chow

**Figure 3:**
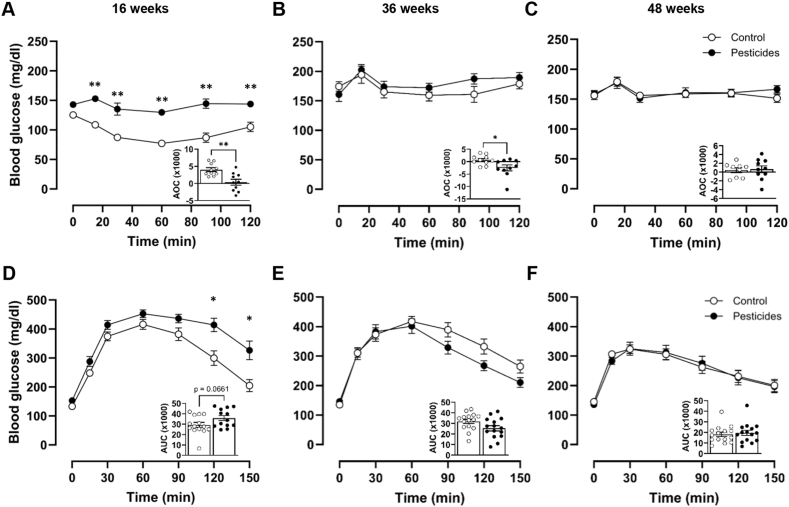
**Insulin and glucose tolerance tests in B6C3H mice exposed or not to pesticides.** (**A, B, C**) Blood glucose levels in B6C3H mice after administration of an intraperitoneal insulin injection (0.75 U/kg BW). Inset shows the area over the curve (AOC) for mice fed control chow (empty circles) or pesticide chow (filled circles). n = 10 mice per group. (**D, E, F**) Blood glucose levels in B6C3H mice after administration of an intraperitoneal (16 weeks) or an oral (36 and 48 weeks) glucose load [1.5 g/kg body weight (BW)]. Inset shows the area under the curve (AUC) for mice fed control chow (empty circles) and those fed pesticide chow (filled circles). n = 13–15 mice per group. Results are presented as mean ± SEM. ∗p < 0.05; ∗∗p < 0.01 compared to mice fed control chow as determined by Student's *t* test (insets) or two-way ANOVA.

Given the obesogenic effect of the pesticide exposure (Fig. 1), we assessed the impact of pesticides on insulin action and glucose homeostasis in B6C3H mice (Figure 3). Mice were subjected to insulin and glucose tolerance tests at different exposure times (16, 36 and 48 weeks). After 16 weeks of exposure, B6C3H mice fed pesticide chow exhibited persistent higher blood glucose following intraperitoneal insulin injection compared to those fed control chow (Figure 3A and inset). Pesticide exposure of B6C3H mice was also associated with a higher HOMA-IR index (Fig. S3A), further supporting insulin resistance. Interestingly, insulin sensitivity was not correlated to body weight increase (Fig. S4A*, linear regression illustrated by the full line*), revealing that pesticide-induced insulin resistance can not be simply explain by body weight differences.

Glucose homeostasis was also investigated by performing glucose tolerance tests. B6C3H mice fed with pesticides displayed higher blood glucose 120 and 150 min after administration of glucose compared to mice fed control chow (Fig. 3D). However, 36 and 48 weeks postexposure, B6C3H mice fed control or pesticide chow diet no longer exhibited differences in insulin sensitivity (Figure 3B–C) nor glucose tolerance (Figure 3E–F), certainly because of the convergence of their body weight after 30 weeks of exposure (Figure 1A,D, E). Overall, our data reveal that B6C3H mice fed the mixture of pesticides for 16 weeks became insulin resistant and developed impaired glucose tolerance.

### 3.4 Insulin resistance and better glucose tolerance in M83 mice exposed to pesticide chow compared to mice fed control chow

**Figure 4:**
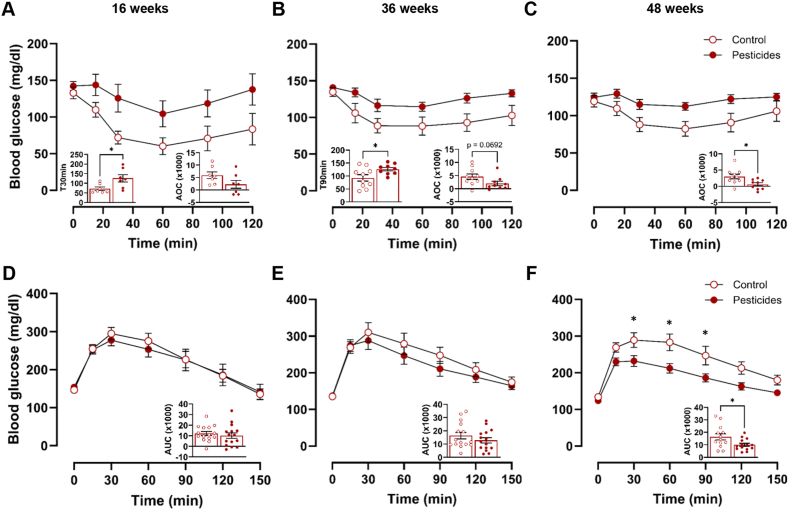
**Insulin and glucose tolerance tests in M83 mice exposed or not to pesticides.** (**A, B, C**) Blood glucose levels in M83 mice after intraperitoneal injection of insulin (0.75 U/kg BW). Inset on right shows the area over the curve (AOC) and inset on left shows blood glucose 30 or 90 min after insulin administration in mice fed control chow (empty circles) and pesticide chow (filled circles). n = 7–10 mice per group. (**D, E, F**) Blood glucose levels in M83 mice after administration of an intraperitoneal (16 weeks) or an oral (36 and 48 weeks) glucose load (1.5 g/kg BW). Inset shows the area under the curve (AUC) for mice fed control chow (empty circles) and pesticide chow (filled circles). n = 15 mice per group. Results are presented as mean ± SEM. ∗p < 0.05 compared to mice fed control chow as determined by Student's *t* test (insets) or two-way ANOVA.

Given the obesogenic effect of pesticides on M83 male mice, similar to that observed in B6C3H mice (Fig. 1), we assessed insulin action and glucose homeostasis in the same way. After 16 and 36 weeks of exposure, M83 mice fed pesticide chow had the tendency to exhibit higher blood glucose following intraperitoneal insulin injection compared to those fed control chow (Figure 4A–B) with significant increased blood glucose levels 30 min (at 16 weeks, Figure 4A, left inset) or 90 min (at 36 weeks, Figure 4B, left inset) after insulin injection. Again, insulin resistance was supported by the higher HOMA-IR index in M83 mice fed pesticide chow for 16 and 36 weeks (Fig. S3A–B). M83 mice fed control or pesticide chow diet for 16 weeks (Fig. 4D) or 36 weeks (Fig. 4E) displayed similar glucose tolerance. After 48 weeks of exposure, M83 mice fed pesticides developed severe insulin resistance as shown by the significant higher blood glucose levels after insulin injection (Figure 4C and inset). Within this group of mice, insulin sensitivity was not correlated with differences in body weight (Fig. S4B, *linear regression illustrated by the full line*), suggesting once again that pesticides may induce insulin resistance through mechanisms independent of body weight. Surprisingly, in contrast to B6C3H and despite reduced insulin sensitivity, M83 mice fed pesticide chow for 48 weeks exhibited a better glucose tolerance than M83 mice fed control chow (Figure 4F and inset).

### 3.5 Enhanced *in vivo* insulin secretion in response to pesticide-induced insulin resistance in M83 mice

**Figure 5:**
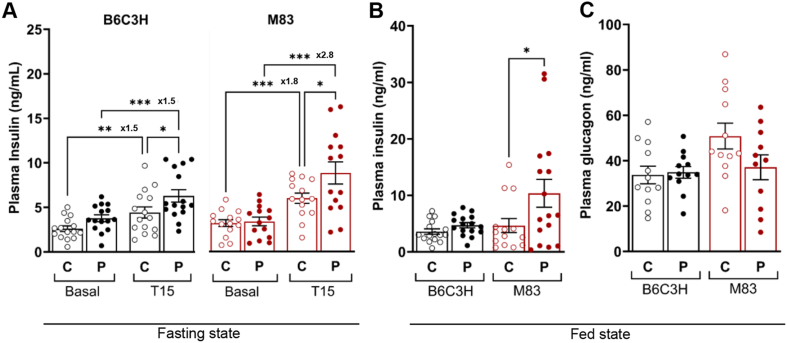
**Plasma insulin and glucagon levels in B6C3H and M83 mice exposed or not to pesticides.** Plasma levels were assessed in B6C3H (in black) and M83 (in red) mice fed control (C) or pesticide (P) chow for 48 weeks (**A**) or 50 weeks (**B, C**). (**A**) Plasma insulin (ng/ml) after 6-h fast (Basal) and 15 min after (T15) administration of an oral glucose load (1.5 g/kg BW). (**B**) Plasma insulin at fed state (ng/ml). (**C**) Plasma glucagon at fed state (ng/ml). n = 11–16 mice per group. Results are presented as mean ± SEM. ∗p < 0.05; ∗∗p < 0.01; ∗∗∗p < 0.001 for indicated comparisons as determined by two-way (**A**) and one-way (**B, C**) ANOVA.

The better glucose tolerance in M83 mice fed pesticide chow observed despite insulin resistance could be caused by increased insulin secretion. Indeed, while M83 mice fed control diet for 48 weeks showed a 1.8-fold increase in plasma insulin levels 15 min after glucose load (Figure 5A, right panel), M83 mice fed pesticide diet displayed a 2.8-fold increase in plasma insulin levels in response to glucose load (Figure 5A, right panel). Of note, B6C3H mice exposed to control or pesticide diet for 48 weeks showed a similar 1.5-fold increase in plasma insulin levels 15 min after glucose load (Figure 5A, left panel). In addition, pesticide exposure increased plasma insulin levels at fed state in M83 mice (Fig. 5B), despite comparable blood glucose levels (Fig. S5). Plasma glucagon levels remained similar in B6C3H and M83 mice exposed to control or pesticide chow (Fig. 5C), and thus could not explain the better glucose tolerance in M83 mice fed pesticides. Overall, these *in vivo* data revealed an unexpected enhanced functional adaptation to increase insulin levels to overcompensate pesticide-induced insulin resistance in the M83 mouse model.

### 3.6 Decreased insulin receptor protein levels in mice exposed to pesticides

**Figure 6:**
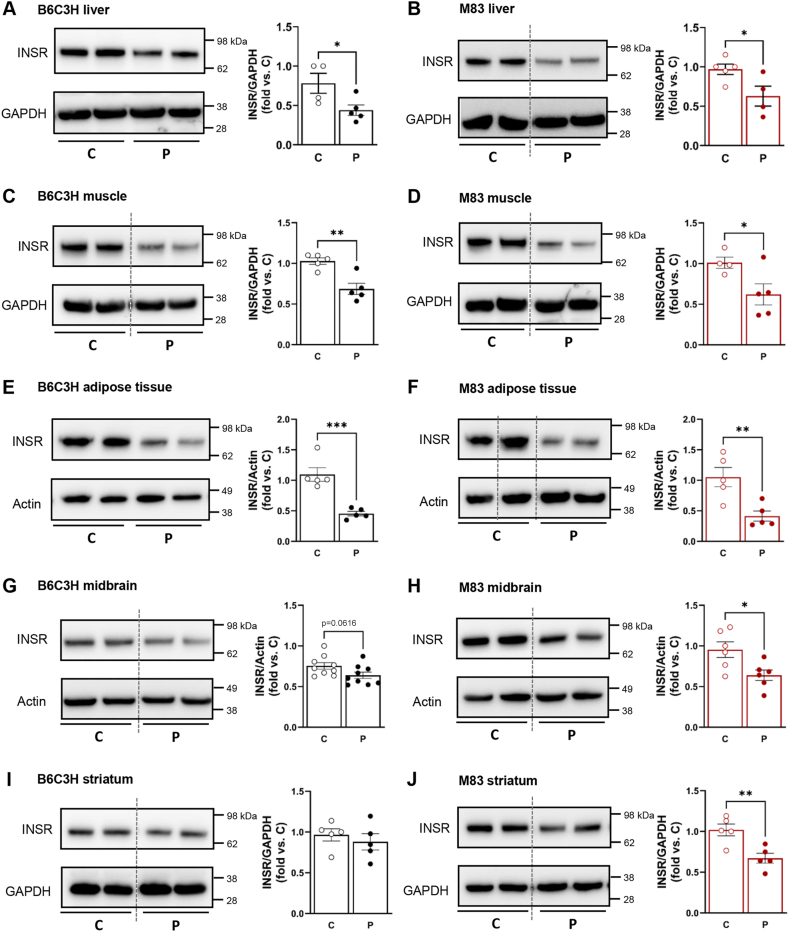
Insulin receptor protein levels in B6C3H and M83 mice exposed or not to pesticides. Protein levels of insulin receptor (INSR) and GAPDH or actin (loading control) were analyzed by western blot in liver (**A, B**), muscle (**C, D**), adipose tissue (**E, F**), midbrain (**G, H**) and striatum (**I, J**) lysates of B6C3H mice (black) and M83 mice (red) fed control (C) or pesticide (P) chow for 26 weeks. Graphs represent the quantification of the blots. n = 4–9 mice per group. For images in **B**, **C**, **D**, **F**, **G**, **H**, **I**, **J** lanes were run on the same gel but were non-continuous (as indicated). Results are presented as mean ± SEM. ∗p < 0.05; ∗∗p < 0.01; ∗∗∗p < 0.001 compared to mice fed control chow as determined by Student's *t* test.

To better characterize the cellular and molecular mechanisms underlying insulin resistance (Figure 3, Figure 4), we explored the main target tissues of insulin action: liver, skeletal muscle and adipose tissue [30]. Given the critical role of the insulin receptor (INSR) in mediating insulin metabolic effects [30], we measured INSR protein levels in these tissues. Overall, INSR protein levels in liver, skeletal muscle and adipose tissue were decreased by 30–60% in both B6C3H (Figure 6A,C, E) and M83 (Figure 6B,D, F) mice fed pesticide chow for 26 weeks.

Since INSR were also found to be expressed in central nervous system, including regions dedicated to motor control, where it may exert neurotrophic and neuroprotective effects [21], we next examined its levels in midbrain and striatum. In M83 mice fed pesticide chow, INSR protein levels were reduced by 34% in midbrain and by 44% in striatum compared to mice fed control chow (Figure 6H,J). In contrast, no significant differences in INSR levels were observed in these regions in B6C3H mice fed pesticide chow (Figure 6G,I), despite peripheral insulin resistance and peripheral reduced expression of INSR.

Altogether, these results suggest that pesticide-induced peripheral insulin resistance could be mediated by decreased INSR protein levels in liver, muscle and adipose tissue in both B6C3H and M83 mice. Moreover, in the central nervous system, pesticide exposure exclusively altered INSR protein levels in midbrain and striatum of M83 mice.

### 3.7 Beta-cell mass adaptation to pesticide-induced insulin resistance in M83 mice

**Figure 7:**
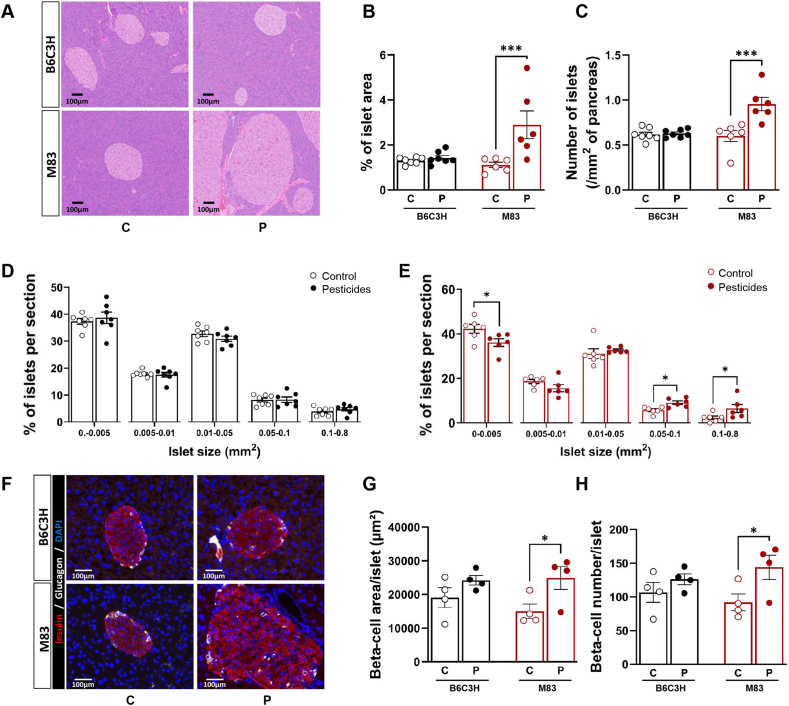
**Histological analysis of endocrine pancreas from B6C3H and M83 mice exposed or not to pesticides. (A)** Representative microphotographs of mouse pancreatic sections stained with H&E, (**B**) percent of section area occupied by islets, (**C**) islet number per mm^2^ of pancreas, (**D, E**) islet distribution per pancreatic section, (**F**) insulin and glucagon staining assessed by immunofluorescence (insulin, red; glucagon, white; nuclei, blue), (**G**) beta-cell area per islet (i.e. insulin-positive area per islet), (**H**) number of beta-cells per islet in B6C3H (black) and M83 (red) male mice fed control chow (C) and those fed pesticide chow (P) for 26 weeks. n = 4–7 mice per group. Results are presented as mean ± SEM. ∗p < 0.05; ∗∗∗p < 0.001 compared to mice fed control chow as determined by two-way ANOVA (**B, C, G, H**) and Student's *t* test (**D, E**).

After exploring the mechanisms related to insulin resistance, we aimed to evaluate whether pancreatic beta-cell mass expansion could account for enhanced plasma insulin levels measured *in vivo* in M83 mice fed pesticide chow (Fig. 5A). For this purpose, islet mass was investigated in pancreases obtained from B6C3H and M83 mice fed control or pesticide chow for 26 weeks. B6C3H mice fed control or pesticide chow showed no differences in islet architecture and islet/beta-cell mass (Figure 7A–G and Fig. S6). By contrast, the percentage of pancreatic section area occupied by islets was increased by 2.3-fold in M83 mice fed pesticide chow in comparison to mice fed control chow (Figure 7A–B and Fig. S6). These results can be explained by an increased number of islets (Fig. 7C), and a modified distribution of islet sizes (decrease of small islets and increase of large islets) (Fig. 7E) in M83 mice exposed to pesticides. Although islet architecture was comparable in pancreases of M83 mice fed pesticide or control chow (Fig. 7F), the surface occupied by beta-cells (Fig. 7G) and their number (Fig. 7H) per islet were significantly increased in M83 mice fed pesticide chow. In contrast the surface occupied by alpha-cells and their number remained unchanged (data not shown). *In vitro* functional analysis further revealed a tendency towards increased glucose-induced insulin secretion in islets isolated from M83 mice exposed to pesticides, although significancies are limited by the low number of samples dedicated to insulin secretion studies (Fig. S7). Overall, our data pointed to a strong expansion of functional beta-cell mass as an overcompensatory mechanism to combat pesticide-induced insulin resistance in M83 mice.

### 3.8 High levels of α-synuclein and enhanced amino acid metabolism in M83 mouse islets

**Figure 8:**
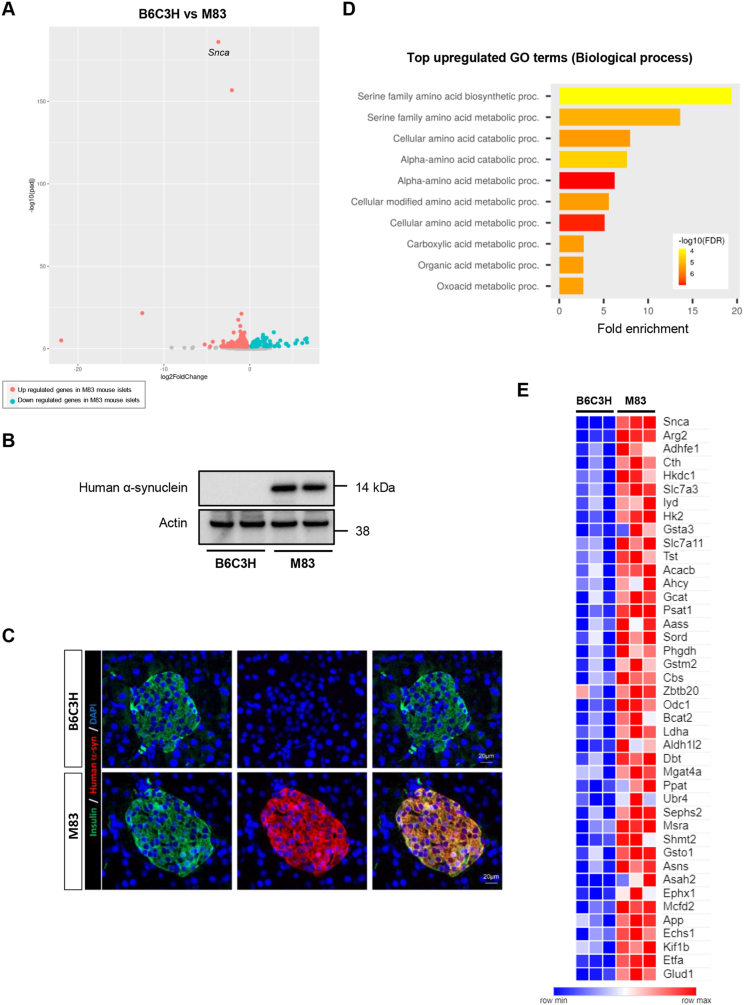
**Gene expression profile in islets isolated from M83 *versus* B6C3H mice.** (**A**) Volcano plot representing differential gene expression between B6C3H and M83 mouse islets. RNA-Seq analysis identified 486 differentially expressed genes, of which *Snca* had the lowest p-value. (**B**) Protein levels of human α-synuclein and actin (loading control) assessed by western blot in islets isolated from B6C3H mice and M83 mice. (**C**) Human α-synuclein immunostaining in B6C3H and M83 mouse pancreatic sections (insulin, green; human α-syn, red; nuclei, blue). (**D**) Gene ontology pathway analysis of the 323 significantly induced genes in M83 mouse islets. The histogram shows the enrichment score for each identified pathways (top 10) in GO terms (Biological process). GO, gene ontology; FDR, false discovery rate. (**E**) Heatmap (gene counts normalized with Relative Log Expression) displaying changes in 42 differentially expressed genes involved in amino acid metabolism (from the GO-Biological process) in islets from B6C3H and M83 mice. n = 3 mice per group.

To investigate the changes in the islet transcriptomic landscape that could be involved in the processes of beta-cell functional compensation in M83 mice exposed to pesticides, RNA-seq analysis were performed. Whereas pesticide exposure did not induce significant changes in islet gene expression in either B6C3H or M83 mice (Fig. S8A–B), transcriptomic analysis pointed to genes differentially regulated between B6C3H and M83 mouse islets (Fig. S8C). Indeed, differential gene expression analysis of the 19,056 genes detected by RNA-seq revealed that the expression of 486 genes was significantly modified in M83 vs. B6C3H mouse islets (p < 0.05). Among the 323 upregulated genes, α-synuclein (*Snca*) was the most significant induced gene in M83 mouse islets in comparison to B6C3H (Figure 8A). Given the high sequence homology between the mouse and human α-synuclein genes [31], transcriptomic analysis did not allow us to determine which of the murine or human form (or both) was actually increased. Evaluation of human and murine α-synuclein protein levels revealed that high levels of human α-synuclein were unequivocally detected in M83 mouse islets by western blot (Fig. 8B) and immunofluorescence (Fig. 8C). Images further showed that human α-synuclein was restricted to islets and not found in acini in pancreatic sections (Fig. 8C). Most likely in relation to the use of the mouse prion protein promoter to drive transgene expression, high expression levels of human α-synuclein found in M83 mouse islet-cells offered the opportunity to investigate its contribution in pancreatic islets. Indeed, functional gene analysis (Gene Ontology) further revealed that upregulated genes in M83 mouse islets were enriched for processes related to amino acid metabolism (Fig. 8D), and 43 of these genes were listed on the heatmap (Fig. 8E). In conclusion, abundant human α-synuclein levels detected in M83 mouse islets [as in neurons (Fig. S9)] was associated with increased expression of genes involved in amino acid metabolism, a process which may account for an enhanced functional adaptation of beta-cells to pesticide-associated insulin resistance.

## 4 Discussion

Whereas the etiology of metabolic and neurodegenerative disorders is multifactorial (genetic component, lifestyle influence, food consumption …), epidemiological studies have linked pesticide exposure to increased risk of obesity, T2D [1,4,32,33] and increased risk of PD [7,[34], [35], [36], [37]]. Some studies suggest the potential role of occupational exposure to persistent (organochlorines) and non-persistent (organophosphates, pyrethroids, triazines) pesticides in the incidence of metabolic disorders [3,[38], [39], [40], [41], [42]] and PD [37], but evidences regarding other classes of pesticides and risks for the general population are more limited. In addition, most experimental studies evaluated the effect of individual compounds, at doses often far from the realistic ones to which the consumers are exposed. To further underscore the interest in studying the effects of the six pesticides selected, toxicological studies and recent publications have demonstrated their accumulation in various metabolic tissues, with all six pesticides and their metabolites accumulating in liver, captan in muscle, boscalid and chlorpyrifos in adipose tissue [43], and both thiacloprid and chlorpyrifos accumulating in brain [43,44].

In our study, the goal was to evaluate the consequences of a pesticide exposure protocol relevant to consumers'exposure. We observed that chronic dietary exposure to a mixture of 6 pesticides at levels below the ADI resulted in body weight gain and insulin resistance in both wild-type mice and those prone to develop PD at late age. The body weight gain observed is in line with our previous study [2] showing that male C57Bl/6J mice exposed to the same diet for 12 months had a greater body weight, and confirms the effects of the pesticide mixture on body weight gain despite some differences in the mouse strains (earlier body weight gain in B6C3H mice and loss of differences with age). Given that food intake was comparable between the groups, increased body weight is more likely caused by pesticide-induced alterations in nutrient absorption, metabolism or energy homeostasis as suggested by the literature [45,46]. Whereas *in vivo* and *in vitro* studies already reported that chronic exposure to a specific pesticide can induce insulin resistance in liver, muscle or adipose tissue [[47], [48], [49], [50]], our results are the first to provide some mechanistic supporting arguments on epidemiological data [1,3,39,40,42,51,52] to demonstrate the effects of a pesticide mixture on insulin resistance *in vivo*. Indeed, the decreased insulin receptor protein levels in the main target tissue of insulin could explain, at least in part, the insulin resistant state observed under such pesticide exposure. Although one might expect that a decrease in insulin receptor levels would impair cellular responsiveness to insulin [30], it remains to determine whether the pesticide cocktail can - either as a consequence or in addition - alter the insulin signaling pathway, such as Akt phosphorylation.

Our data further indicate a glucose intolerance state in B6C3H male mice exposed to pesticides for 16 weeks. The later further support our observations [2], with some differences most likely related to the mouse strain (unexpected early glucose intolerance onset and loss of differences with age). Nevertheless, our *in vivo* study points to overweight, insulin resistance and glucose intolerance in wild-type mice exposed to pesticides via the diet for 16 weeks. It is important to note that differences in insulin sensitivity between B6C3H exposed and non-exposed mice were not seen at 36 and 48 weeks since non-exposed B6C3H mice also became insulin resistant with age. Whereas the molecular mechanisms responsible for this pre-diabetic state observed at 16 weeks were not fully investigated, our pancreas analyses suggest that B6C3H male mice failed to adequately adapt beta-cell mass and insulin secretion to face insulin resistance induced by pesticides.

M83 mice exposed to pesticides developed early motor deficits beginning at 12 weeks of exposure, without reaching the typical neuropathology and paralysis occurring beyond the age of 22 months [22]. Although no typical molecular characteristics of PD were apparent, significant decrease in INSR levels was observed in midbrain and striatum of these mice. Given the potentially neurotrophic and neuroprotective effects of insulin [53], we could speculate that alteration of its signaling induced by pesticides could participate to the locomotor disorders observed in M83 mice. These data are particularly interesting since a decrease in INSR were reported in the *substantia nigra* of human subjects with PD [21] and specific invalidation of the INSR in mouse brain led, among other things, to impaired dopaminergic function [53]. In addition, decreased INSR levels were observed in insulin-sensitive tissue of M83 mice exposed to pesticides. The associated peripheral insulin resistant state observed in our model may also contribute to motor alterations, as already demonstated in another α-synucleinopathy transgenic mouse model made obese and insulin resistant by a high-fat diet [54].

Insulin-resistant M83 mice surprisingly showed improved glucose tolerance after 48 weeks of pesticide exposure. This is accompanied by an enhanced *in vivo* insulin secretion due, at least in part, to an increase of functional beta-cell mass (islet size, number of beta-cells/islet). Whereas the mechanisms involved in such beta-cell mass expansion remain unknown, high expression levels of α-synuclein in M83 mouse islets as well as its described roles in beta-cell function could provide some explanation for the functional adaptation. Indeed, homozygous mice overexpressing human α-synuclein were reported to display improved glucose tolerance and enhanced glucose-induced insulin secretion [55]. On the contrary, α-synuclein knockout mice were glucose intolerant with impaired glucose-stimulated insulin secretion [18,55]. Despite few studies pointing to an inhibitory role of α-synuclein on insulin secretion [17,18], our data are more in line with a physiological role of α-synuclein in exocytosis-related processes (vesicle clustering and docking, SNARE complex assembly) shared and common to the regulation of neurotransmission [56] and insulin secretion. As the mice used in this study were hemizygous for the expression of human α-synuclein, it is not surprising that its positive role on beta-cell function is only revealed in cases of high insulin demand caused by pesticide-induced insulin resistance.

In addition, the large scale strategy used in this study revealed an unexpected transcriptional upregulation of genes involved in amino acid metabolism in islets overexpressing α-synuclein. Given the prominent adaptive capacity of these islets to overcompensate for pesticide-induced insulin resistance, this upregulation may be a critical mechanism. In line with this speculation, successful adaptation to the increased metabolic demand was shown to be associated with upregulation of amino acid metabolism in a diabetes-resistant obese mouse model [57]. Indeed, amino acids are the building blocks of proteins, and their metabolism is essential to fuel the hyperactive biosynthetic machinery of beta-cells in a context of increased metabolic demand (i.e. proinsulin biosynthesis, gene expression, beta-cell mass expansion, and even insulin secretion [58,59]).

Interestingly, metabolic changes revealed by transcriptomics in islets of mice prone to develop PD were also identified by metabolomics in the brain and serum of PD rodent models at early stages, as well as in PD patients [60]. In fact, several amino acid metabolic processes, including serine, were increased at early stages of PD in rats, and may possibly protect them from developing motor symptoms [58,[60], [61], [62]]. Overall, modified amino acid metabolism in islets and brain could underpin common favorable conditions for compensatory mechanisms (i.e. mitochondrial function, endoplasmic reticulum homeostasis, cell trophicity), thus highlighting again some shared processes in the early stages of PD and T2D. While studies on PD rodent models [our and [60]] suggest the possible involvement of α-synuclein in such metabolic changes in both neurons and islets, it remains to be assessed whether α-synuclein is directly involved in this genic/epigenetic regulation, as suggested in different contexts [63,64].

Overall, our study demonstrates that dietary exposure to a mixture of pesticides at levels below the ADI leads to body weight gain and insulin resistance in both wild-type and mice prone to develop PD. Insulin resistance was explained by a decreased INSR expression in insulin-sensitive tissues. However, only M83 mice exposed to pesticides showed early locomotor deficits associated with decreased expression of INSR in midbrain and striatum. Whereas wild-type mice are glucose intolerant, M83 mice surprisingly display improved glucose tolerance after 12 months of exposure. This is accompanied by an increase in beta-cell mass associated with enhanced insulin secretion *in vivo*. The abundant expression of α-synuclein in M83 mouse islets, revealed by transcriptomics, is associated with an enrichment of genes involved in amino acid metabolism. This study therefore points to an unexpected role of α-synuclein in amino acid metabolism, likely to explain beta-cell adaptation in the face of pesticide-induced insulin resistance.

## CRediT authorship contribution statement

**Ambrine Arrar:** Writing – original draft, Visualization, Validation, Methodology, Investigation, Formal analysis, Conceptualization. **Nour Mesto:** Validation, Methodology, Investigation, Formal analysis. **Roxane Descaillot:** Validation, Investigation, Formal analysis, Conceptualization. **Muris Humo:** Writing – review & editing, Validation, Methodology, Investigation, Formal analysis. **Aude De Cesar:** Methodology, Investigation. **Damien Gaillard:** Methodology, Investigation. **Latifa Lakhdar:** Supervision, Investigation. **Johann Vulin:** Writing – review & editing, Validation, Methodology, Investigation, Formal analysis. **Claire Aufauvre:** Investigation, Formal analysis. **Raphaëlle Baumier:** Investigation. **Camille Bellières:** Validation, Resources, Methodology, Investigation, Formal analysis. **Jacques Imbert:** Validation, Resources, Methodology, Investigation, Formal analysis. **Prunelle Perrier:** Investigation, Formal analysis. **Emilien L. Jamin:** Validation, Resources, Methodology, Investigation, Formal analysis. **Romain Vuillaume:** Validation, Resources, Methodology, Investigation, Formal analysis. **Jean-Noël Arsac:** Writing – review & editing, Project administration, Conceptualization. **Anne Fougerat:** Writing – review & editing, Formal analysis. **Magalie A. Ravier:** Writing – original draft, Validation, Supervision, Resources, Methodology, Investigation, Formal analysis, Conceptualization. **Thierry Baron:** Writing – review & editing, Validation, Supervision, Resources, Project administration, Methodology, Funding acquisition, Conceptualization. **Laurence Gamet-Payrastre:** Writing – review & editing, Validation, Supervision, Resources, Project administration, Methodology, Investigation, Funding acquisition, Formal analysis, Conceptualization. **Safia Costes:** Writing – original draft, Validation, Supervision, Resources, Project administration, Methodology, Investigation, Funding acquisition, Formal analysis, Conceptualization.

## Declaration of competing interest

The authors declare that they have no known competing financial interests or personal relationships that could have appeared to influence the work reported in this paper.

## Data Availability

Data will be made available on request.
