# Supplementary material for: Insulin resistance induced by pesticides is overcome by pancreatic islet adaptation in a mouse model of Parkinson's disease
Source: Mol Metab. 2026 May 26;109:102388. doi: 10.1016/j.molmet.2026.102388 (PMC13273497; doi:10.1016/j.molmet.2026.102388)
Supplement: Multimedia component 1 [file mmc1.docx]

**Supplemental Material**

**Insulin resistance induced by pesticides is overcome by pancreatic islet adaptation in a mouse model of Parkinson’s disease**

Ambrine Arrar, Nour Mesto, Roxane Descaillot, Muris Humo, Aude De Cesar, Damien Gaillard, Latifa Lakhdar, Johann Vulin, Claire Aufauvre, Raphaëlle Baumier, Camille Bellières, Jacques Imbert, Prunelle Perrier, Emilien L. Jamin, Romain Vuillaume, Jean-Noël Arsac, Anne Fougerat, Magalie A. Ravier^#^, Thierry Baron^*^, Laurence Gamet-Payrastre^*^, Safia Costes*^#^

**Table of Contents**

Section 1: Supplemental Methods

Fig. S1 Quantity of ingested pesticides per gram of BW mouse.

Fig. S2 Food intake of B6C3H and M83 mice exposed or not to pesticides.

Fig. S3 Homeostasis model for insulin resistance for B6C3H and M83 mice exposed or not to pesticides.

Fig. S4 Relationships between insulin sensitivity *versus* the body weight of B6C3H and M83 mice exposed or not to pesticides.

Fig. S5 Blood glucose at fed state in B6C3H and M83 mice exposed or not to pesticides.

Fig. S6 Microphotographs of mouse pancreatic sections from B6C3H and M83 mice exposed or not to pesticides.

Fig. S7 Glucose-induced insulin secretion in islets isolated from B6C3H and M83 mice exposed or not to pesticides.

Fig. S8 Mean Average (MA) plots representing gene expression dataset of islets isolated from B6C3H and M83 mice exposed or not to pesticides.

Fig. S9 Human and murine α-synuclein protein levels in M83 and B6C3H mouse striatum.

Table S1 Chemical families, functions, and acceptable daily intake (ADI) (mg/kg body weight/day) of each pesticide, and the expected and measured pesticides concentrations (determined level) (µg/kg food) in the animal pellets.

Table S2 Antibodies used for Western blot (WB) and Immunofluorescence (IF).

References References for work cited in the supplemental material

**SUPPLEMENTAL METHODS**

**Dopaminergic neuron count**

To determine the level of neurodegeneration within the *substantia nigra pars compacta* (SNpc) we performed the unbiased stereological estimation method [1]. We adapted the method from previously published works [2] (Mironov et al., 2020).

After euthanasia, mice were perfused transcardially with 15 mL of phosphate-buffered saline (PBS) using a 25-G needle and an infusion pump, followed by 50 mL of 4% paraformaldehyde (PFA) in PBS. After being removed, brain was immersed overnight at +4°C in 4% PFA solution for post-fixation treatment. After PBS rinse steps, the mouse brain was dissected in the coronal plane at the region comprised between -2 mm and -4 mm from bregma (Paxinos and Franklin, mouse brain atlas). The central macro-section of the brain, including the SNpc, was immersed in 30% sucrose/PBS solution for cryo-protection and incubated up to 2 days at 4 °C. Then, the SNpc macro-section was placed into a cryomold filled with optimal cutting temperature (OCT) compound and frozen on dedicated area in cryostat apparatus (Leica CM 1860 UV). At this step, molded macro-sections could be conserved for several months at -80°C. We performed 30 µm serially cryo-sections in order followed by use of 12-wells culture plates. After one series of 12 cryo-sections, the next one was placed in the first well, followed by subsequent cryo-sections in following wells. Finally, the 4 column’s plates represent 4 series of cryo-sections included in the SNpc.

For immunohistochemical tyrosine hydroxylase (TH) staining, we selected one series of sections per mouse (1:4 sampling proportion); all following steps were carried out in free-floating conditions. Firstly, under agitation, selected series of sections were incubated 5 minutes in a 3% H2O2 solution to avoid endogen peroxidase reaction. This step was followed by 30 minutes incubation in 10% normal goat serum (NGS)/2% bovine serum albumin (BSA)/0.5% Triton100 detergent in PBS. Then, we applied a rabbit anti-mouse TH (1:2000 dilution) (Abcam, #Ab112) antibody diluted in 2% NGS/2% BSA/0.5% detergent in PBS overnight at 4 °C. After PBS rinse steps, horseradish peroxidase (HRP) coupled secondary antibodies against rabbit immunoglobulin (1:1000 dilution) (Clinisciences, #4010-05) for 2 h at room temperature was applied. Finally, staining was carried out thanks to 3 minutes incubation in a solution of 3,3-diaminobenzidine-tetrahydrochloride (DAB) and peroxidase substrate (Vector Laboratories, #SK 4105, ImmPACT® DAB Substrate Kit, Peroxidase). Sections were mounted with non-aqueous mounting medium (ThermoScientific, #4112).

Images were acquired using a confocal scanner (Yokogawa CQ1). After framing the fields containing the SNpc in x and y, the center of each slice was determined using Z focus. From the latter, 2 µm-thick optical sections were made above and below this central section, to obtain a 22 µm-thick captured sample. The following steps were carried out using image J software and the toolkit developed by Mironov (Mironov et al., 2020). Briefly, the 11 optical slices were flattened using the maximum intensity method, thereby representing all the labeled neurons in the slice. The counting frame was then applied to the image with a random start anchor. Neurons present in the frame were counted manually.

Concerning the statistical analysis of the data, first the normal distribution of the data was verified using a Shapiro-Wilk test, then the equality of variances was evaluated using Fisher's test to determine whether or not the Welsh correction on the Student's *t* test comparing the means is necessary.

**Mouse islet isolation and gene expression studies**

Pancreatic islets were isolated after collagenase digestion of pancreases [3,4] obtained from B6C3H and M83 male mice fed control chow for 50 weeks. Total RNA was extracted and DNase-treated using the RNAeasy Mini Kit (Qiagen) according to the manufacturers’ instructions. RNAs concentration and purity/quality were evaluated using the NanoDrop™ 1000 (Thermo Scientific). The following procedures were then performed by the MGX platform. The Stranded mRNA prep ligation kit was used to prepare RNA-seq libraries (Illumina, San Diego, CA, USA) according to the manufacturer's instructions. Briefly, polyadenylated RNAs were selected using oligo-dT magnetic beads, fragmented using divalent cations at elevated temperature and reverse transcribed using random hexamers, reverse transcriptase and actinomycin D. Deoxy-TTP was replaced by dUTP during the second strand synthesis to prevent its amplification by PCR. Double stranded cDNAs were adenylated at their 3' ends and ligated to Illumina's pre-index anchors. Ligated cDNAs were PCR amplified for 12 cycles with primers including unique dual indexes (UDI) and the PCR products were purified using AMPure XP Beads (Beckman Coulter Genomics, Brea, CA, USA). The size distribution of the resulting libraries was monitored using a Fragment Analyzer (Agilent Technologies, Santa Clara, CA, USA) and the libraries were quantified using the KAPA Library quantification kit (Roche, Basel, Switzerland). The libraries were denatured with NaOH, neutralized with Tris-HCl, and diluted to 90 pM. Clustering and sequencing were performed on a NovaSeq 6000 (Illumina, San Diego, CA, USA) using the single read 100nt protocol on 1 lane of a flow cell SP.

Image analysis and base calling were realized in real-time using the NovaSeq Control Software and the Real-Time Analysis 3 software respectively (Illumina, San Diego, CA, USA). Demultiplexing and FASTQ files generation were carried out using Illumina’s bclConvert software (v4.2.4). The quality control of raw data and demultiplexed reads was assessed using respectively Illumina’s Sequencing Analysis Viewer (SAV) software and the FastQC (v0.12.1) software from the Babraham Institute. Contaminant screening was performed using FastQ Screen (v0.15.3, Babraham Institute). Adapters were trimmed using trim_galore (v0.6.10), himself using cutadapt (v4.2). The alignment program HiSat2 (v2.2.1) [5,6] was used to align the reads to the mouse genome (GRCm39), which index was built (hisat2-build) using a set of gene model annotations [gff file downloaded from ENSEMBL (release 109) the 2023-02-15]. For alignment, HiSat2 option -rna-strandness- has been set to RF, in accordance with the kit used for library construction. The option -avoid-pseudogene- has also been used, in order to try and avoid aligning to pseudogenes. Otherwise, default HiSat2 options have been used.

Samtools (v1.18) was used to sort and index the alignment files. Then, gene counting was performed with Featurecounts 2.0.6 [7]. As the data is from a strand-specific assay, the reads have to be mapped to the opposite strand of the gene (-s 2 option). Before statistical analysis, genes with less than 15 reads (cumulating all the analysed samples) were filtered out.

Differentially expressed genes were identified using the Bioconductor [8] package DESeq2 1.38.3 [9] (R version 4.2.2). Data were normalized using the DESeq2 normalization method. Genes with adjusted p-value below 5% (according to the FDR method from Benjamini-Hochberg) were called differentially expressed. Pathway analyses (Gene Ontology (GO) term enrichment) of differentially upregulated genes were performed using ShinyGO v0.80 [10] with all genes considered in the DGE as background. The heatmap was generated using the Morpheus online tool on the basis of relative gene expression levels. Each row represents a gene, and each column represents a mouse islet sample.

**Homeostasis model of insulin resistance (HOMA-IR)**

The HOMA-IR index was calculated as [6h-fasting glucose (mg/dl) × 6h-fasting serum insulin (ng/ml)/405], with lower values indicating a higher degree of insulin sensitivity.

**Insulin secretion from isolated islets**

Freshly isolated mouse islets were preincubated for 1 h in KRB supplemented with 2.8 mM glucose before being distributed in batches of 5 islets for the incubation (in triplicate). Islets were incubated for 1 h at 37°C in 800 μL of KRB containing 16.7 mM glucose. Supernatants were collected and islets were harvested in 150 μL acid ethanol for insulin content. Insulin release and contents were measured by Homogenous Time-Resolved Fluorescence (HTRF) (Insulin ultra sensitive kit, Revvity/Cisbio) according to the manufacturer’s instructions. HTRF signals were measured using Pherastar FS (BMG Labtech) microplate reader. Insulin release was normalized to insulin content.


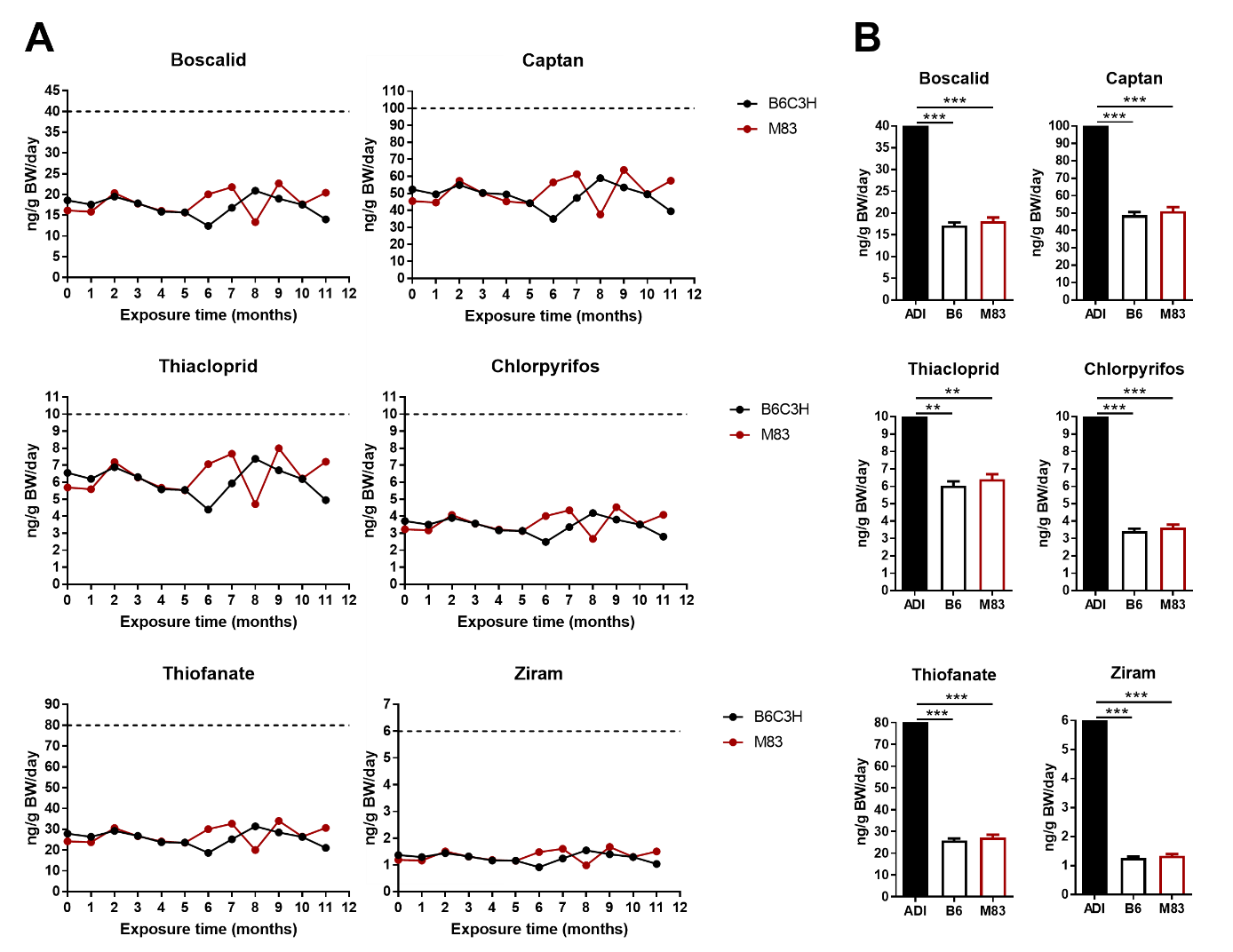


**Fig. S1. Quantity of ingested pesticides per gram of mouse BW.** (**A**) Level of exposure based on exact pesticide levels in pellets and measured food consumption as a function of exposure duration. Dotted lines illustrate ADI. (**B**) Mean pesticide exposure in B6C3H (B6) and M83 (M83) mice. Results are presented as mean ± SEM; **p<0.01; *** p<0.001 as determined by one-way ANOVA. n=7 mice per group.


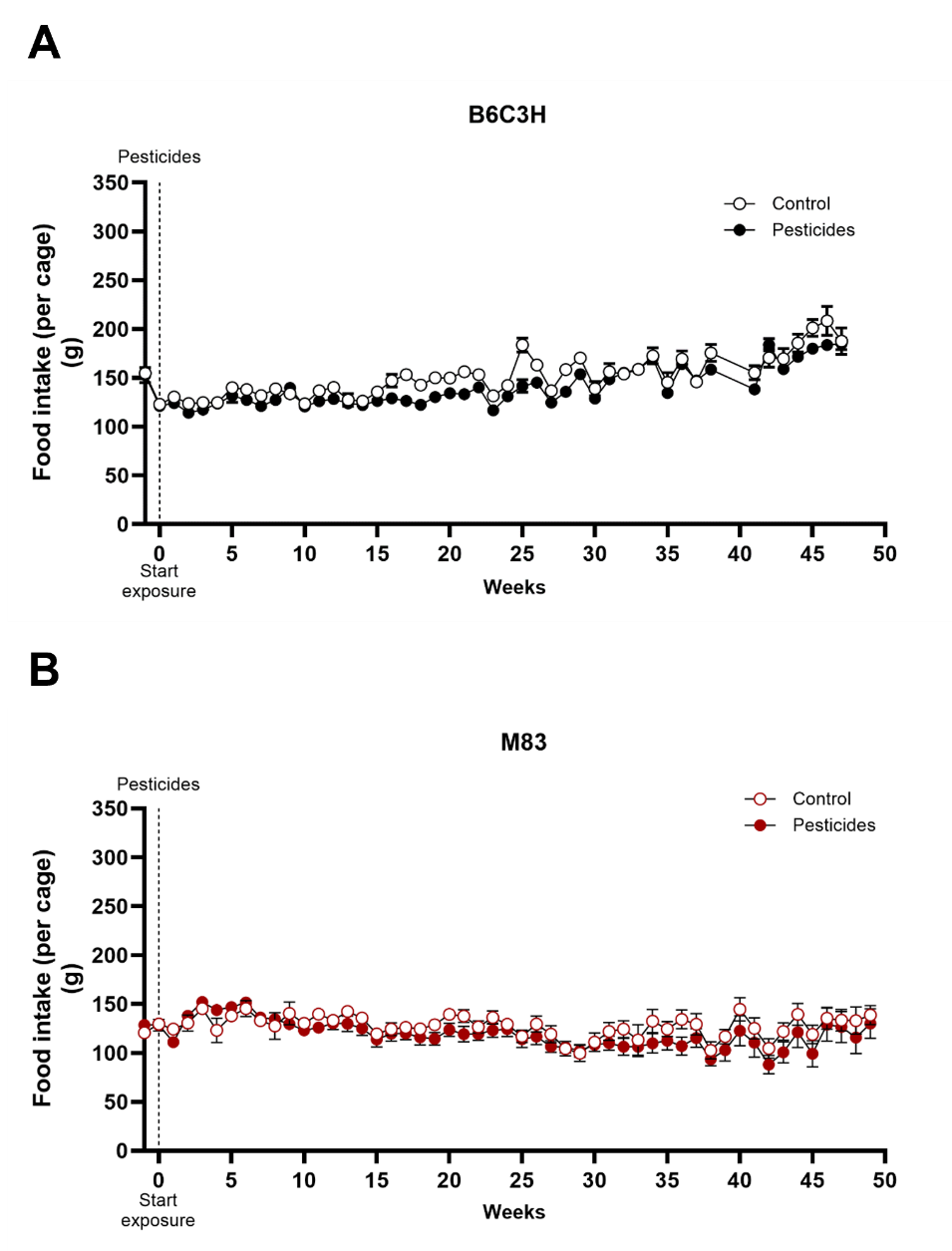


**Fig. S2.** **Food intake of B6C3H and M83 mice exposed or not to pesticides.** Food intake was assessed for B6C3H (**A**, black) and M83 (**B**, red) mice. Graphs show food intake (g) per cage for mice fed control (empty circles) or pesticide (filled circles) chow and from 2 weeks prior exposure to 50 weeks. Data are presented as mean ± SEM. n=23-35 mice per group.


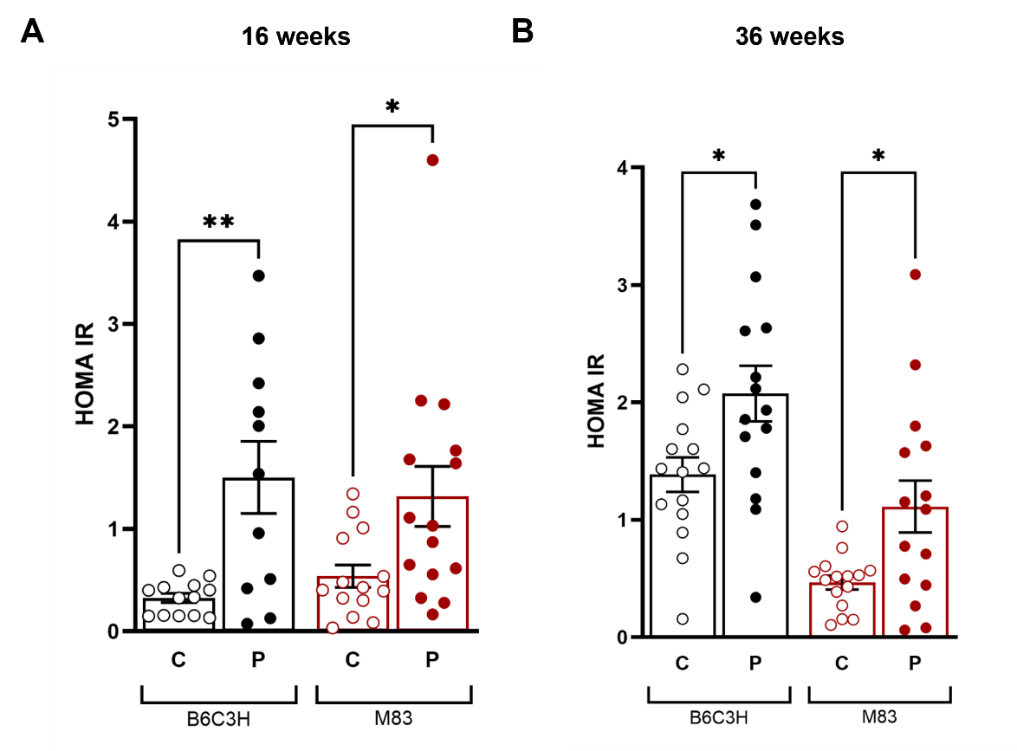


**Fig. S3.** **Homeostasis model for insulin resistance for B6C3H and M83 mice exposed or not to pesticides.** Homeostasis model for insulin resistance (HOMA-IR) index was calculated for B6C3H (black) and M83 (red) mice fed control (C) and pesticide (P) chow for 16 weeks (**A**) and 36 weeks (**B**), as described in the Supplemental Methods section. n=13-15 mice per group. Results are presented as mean ± SEM. *p<0.05; **p<0.01 for indicated comparisons as determined by one-way ANOVA.


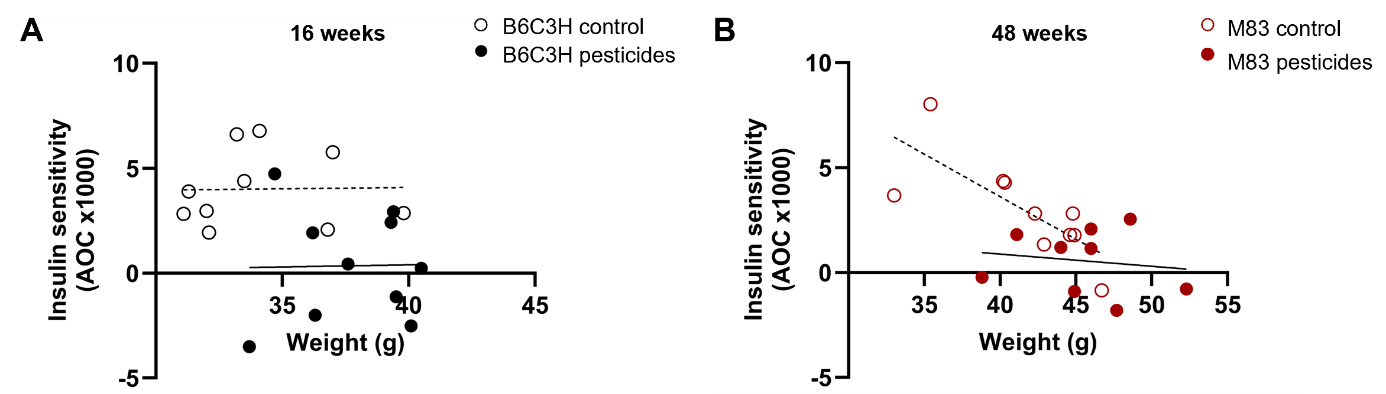


**Fig. S4.** **Relationships between insulin sensitivity *versus* the body weight of B6C3H and M83 mice exposed or not to pesticides.** (**A**) Relationship between insulin sensitivity (expressed in AOC for plasma glucose concentrations during the ITT) vs. the body weight of B6C3H male mice fed control chow (empty circles) and those fed pesticide chow (filled circles) for 16 weeks. The dotted line represents the linear regression for B6C3H mice fed control chow (r=0.2, p=0.58) and the full line represents the linear regression for B6C3H mice fed pesticides chow (r=-0.09, p=0.81). (**B**) Relationship between insulin sensitivity (expressed in AOC for plasma glucose concentrations during the ITT) vs. the body weight of M83 male mice fed control chow (empty circles) and those fed pesticide chow (filled circles) for 48 weeks. The dotted line represents the linear regression for M83 mice fed control chow (r=-0.83, p=0.005) and the full line represents the linear regression for M83 mice fed pesticides chow (r=-0.06, p=0.89). n=9-10 mice per group. Spearman correlation analyses were used to evaluate the relationships.


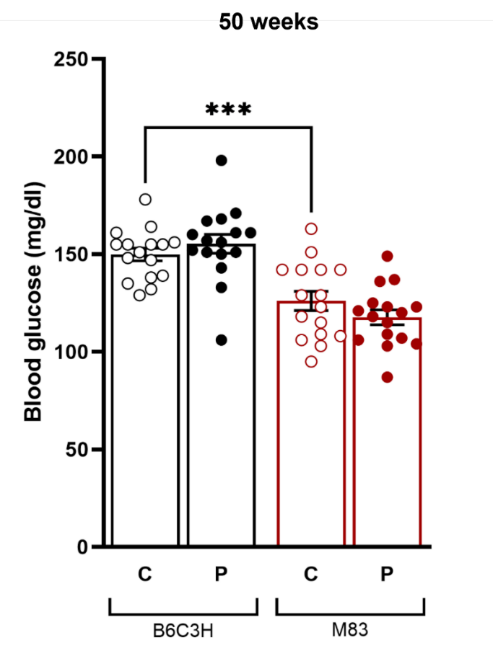


**Fig. S5.** **Blood glucose at fed state in B6C3H and M83 mice exposed or not to pesticides.** Blood glucose at fed state (mg/dl) in B6C3H (black) and M83 (red) mice fed control (C) or pesticide (P) chow for 50 weeks. n=16 mice per group. Results are presented as mean ± SEM. ***p<0.001 for indicated comparisons as determined by one-way ANOVA.


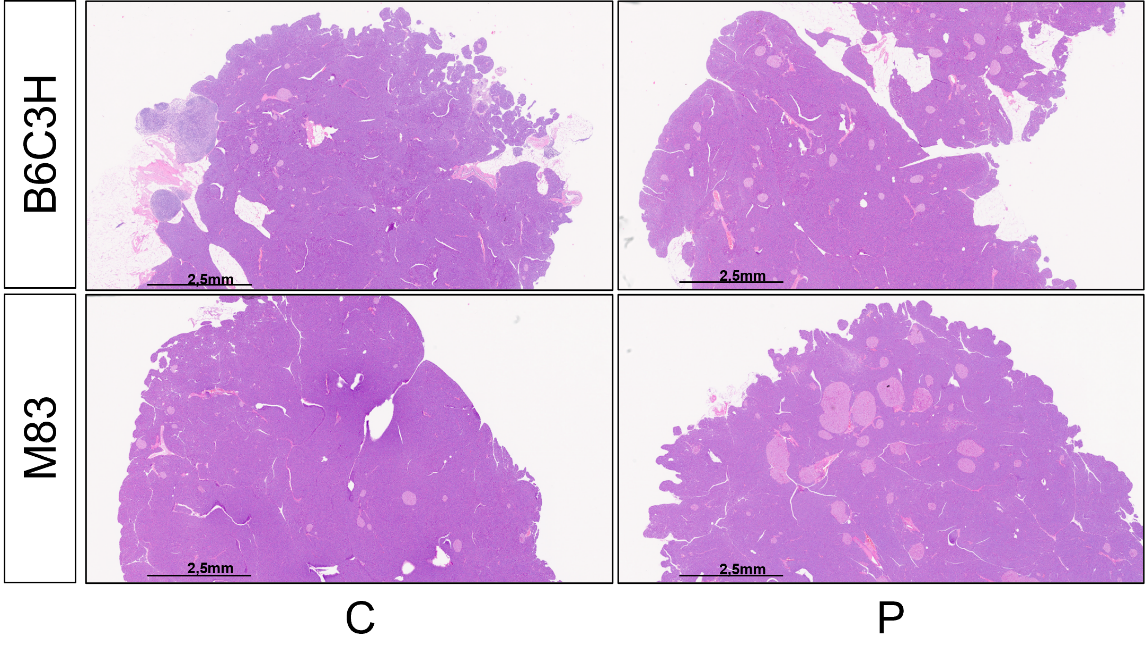


**Fig. S6.** **Microphotographs of mouse pancreatic sections from B6C3H and M83 mice exposed or not to pesticides.** Representative microphotographs of mouse pancreatic sections from B6C3H and M83 mice fed control (C) or pesticide (P) chow for 26 weeks and stained with H&E.


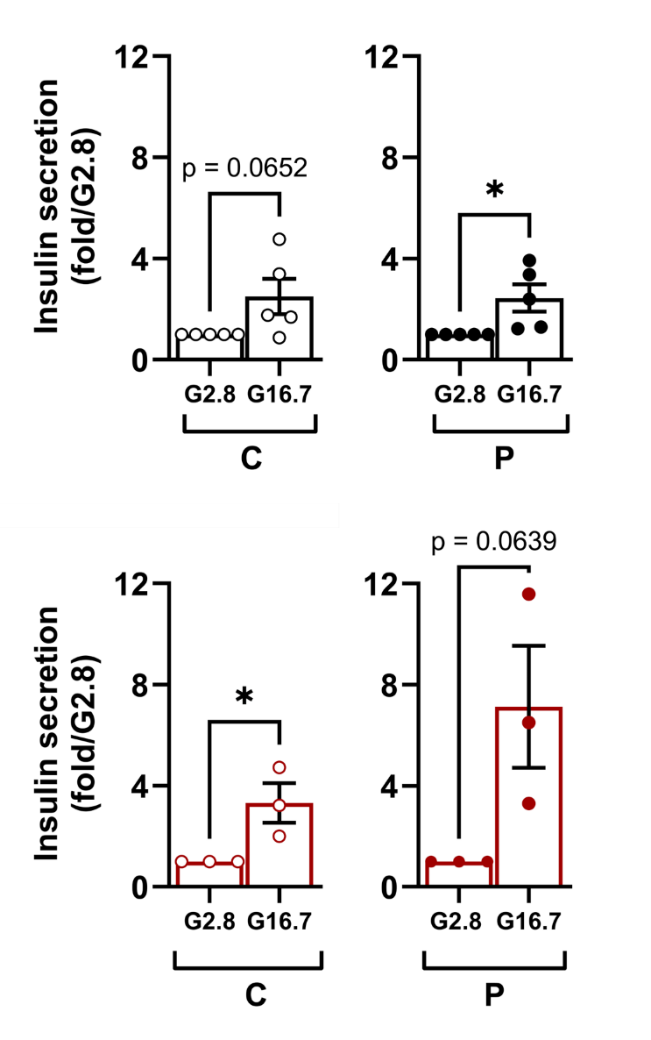


**Fig. S7.** **Glucose-induced insulin secretion in islets isolated from B6C3H and M83 mice exposed or not to pesticides.** Islets were isolated from B6C3H (black) and M83 (red) mice fed control (C) or pesticide (P) chow for 50 weeks. Following 1h quiescent period in Krebs 2.8 mM glucose (G2.8), islets were stimulated with 16.7 mM glucose (G16.7) for 1h at 37 °C. The graph represents insulin secretion normalized to insulin content. n=3-5 mice per group. Results are presented as mean ± SEM. *p<0.05 as determined by Student’s *t* test.


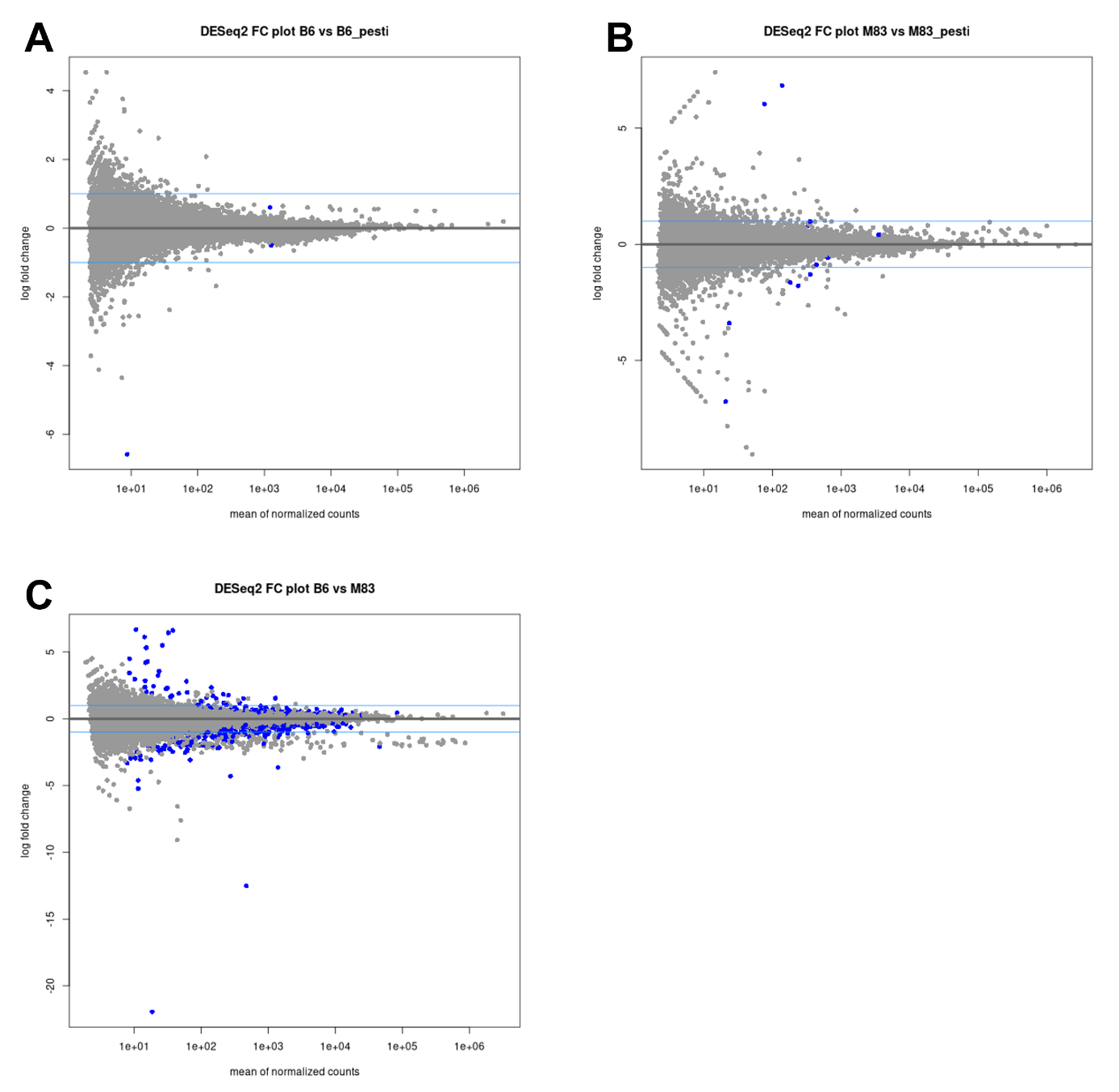


**Fig. S8. Mean Average (MA) plots representing gene expression dataset of islets isolated from B6C3H and M83 mice exposed or not to pesticides.** (**A**) MA plot of islet dataset comparing non-exposed B6C3H mice *versus* B6C3H mice exposed to pesticides for 50 weeks. Each dot represents one gene, and the blue dots indicate the 3 genes identified to be differentially expressed. (**B**) MA plot of islet dataset comparing non-exposed M83 mice *versus* M83 mice exposed to pesticides for 50 weeks. The blue color indicates the 12 genes identified to be differentially expressed. (**C**) MA plot of islet dataset comparing non-exposed B6C3H mice *versus* M83 mice. The blue dots indicate the 486 genes identified to be differentially expressed. The x-axis shows the mean of normalized counts, while y-axis shows the log2(fold). Genes with significant expression differences (adjusted p-value<0.05, calculated using DESeq2) are shown in blue. n=3 mice per group.


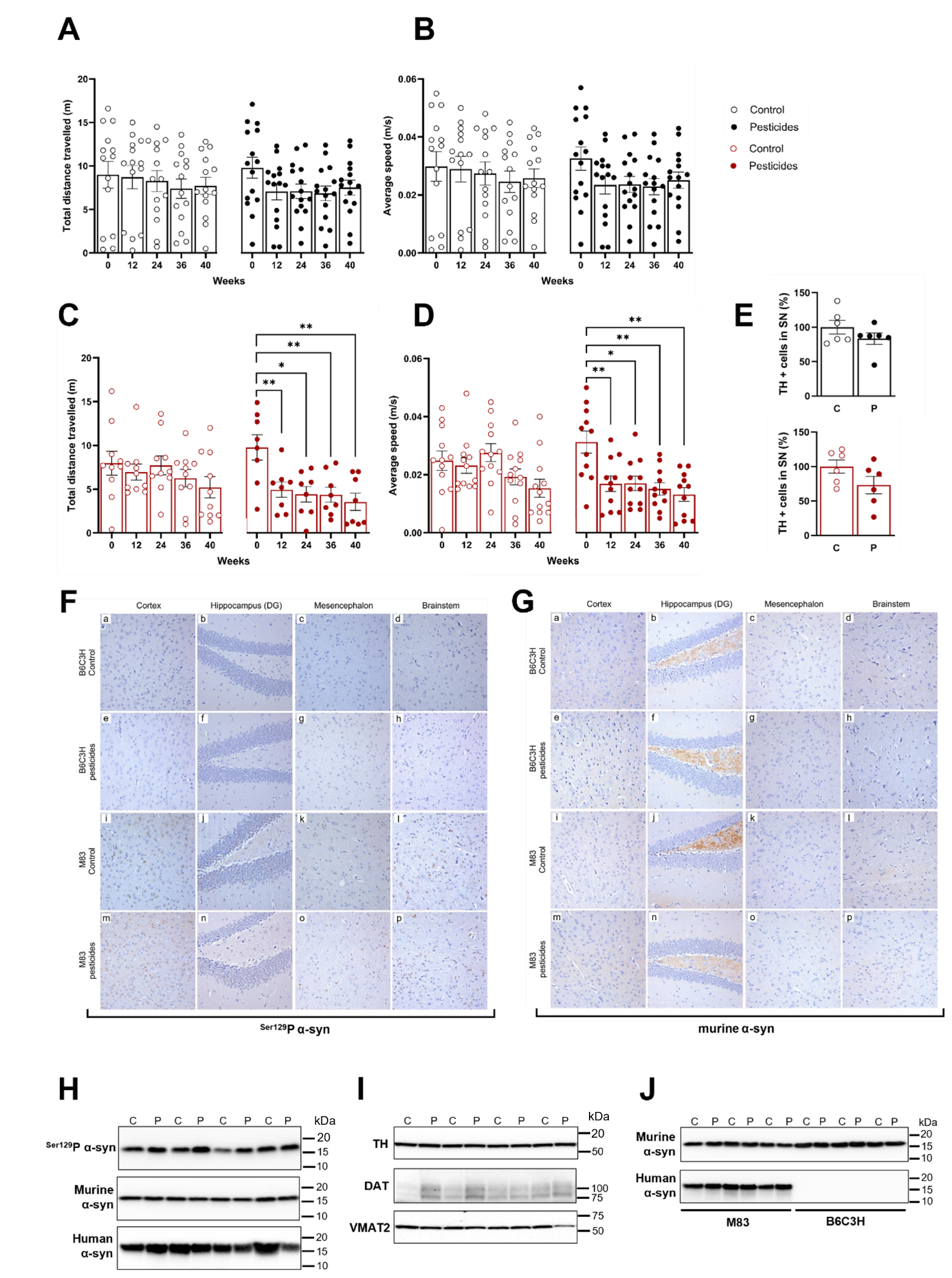


**Fig. S9. Human and murine α-synuclein protein levels in M83 and B6C3H mouse striatum.** Western blot detection of human and murine α-synuclein in striatum of B6C3H and M83 mice exposed (P) or not (C) to pesticides. n=3 mice/group.

| **Pesticide** | **Chemical family** | **Function** | **ADI**  **(mg/kg BW/day)** | | **Expected quantity**  **(µg/kg food)** | **Determined level**  **(µg/kg food)** |
| --- | --- | --- | --- | --- | --- | --- |
| Boscalid | Carboxamide | Fungicide | | 0.04 | 240 | 190 ± 0.028 |
| Captan | Dicarboximide | Fungicide | | 0.1 | 600 | 535 ± 134 |
| Chlorpyrifos | Organophosphorus | Insecticide | | 0.01 | 60 | 38 ± 4.9 |
| Thiacloprid | Neonicotinoid | Insecticide | | 0.01 | 60 | 67 ± 38 |
| Thiofanate | Benzimidazole | Fungicide | | 0.08 | 480 | 285 ± 120 |
| Ziram | Dithiocarbamate | Fungicide | | 0.006 | 36 | 14 ± 6 |

Note: BW, Body weight; ADI, Acceptable daily intake (<https://ephy.anses.fr/>).

Expected quantity refers to the incorporated quantities of pesticides in mice pellets.

**Table S1.** Chemical families, functions, and acceptable daily intake (ADI) (mg/kg BW/day) of each pesticide and the expected and measured pesticides concentrations (determined level) (µg/kg food) in the animal pellets.

| **Application** | **Antibody** | **Host** | **Source** |
| --- | --- | --- | --- |
| **WB** | INSR (IRβ) | Rabbit | #3025T, Cell Signaling |
|  | GAPDH | Rabbit | #2818S, Cell Signaling |
|  | β-Actin | Rabbit | #CS4970, Cell Signaling |
|  | Human α-synuclein | Rabbit | #ab138501, Abcam |
|  | Murine α-synuclein | Rabbit | #D37A6, Cell Signaling |
| **IF** | Insulin | Guinea pig | #PA1-26938, Invitrogen |
|  | Glucagon | Mouse | #G2654, Sigma-Aldrich |
|  | Human α-synuclein | Rabbit | #ab138501, Abcam |
|  | Alexa Fluor 488 AffiniPure | Donkey anti-rabbit | #711-545-152, Jackson immunoresearch |
|  | Cy3-affiniPure | Donkey anti-guinea pig | #706-165-148, Jackson immunoresearch |
|  | Alexa Fluor 680 AffiniPure | Goat anti-mouse | #115-625-146, Jackson immunoresearch |
|  | Alexa Fluor 488 AffiniPure | Donkey anti-guinea pig | #706-546-148, Jackson immunoresearch |

**Table S2.** Antibodies used for Western blot (WB) and Immunofluorescence (IF).

**REFERENCES**

1. West MJ, Slomianka L & Gundersen HJ. Unbiased stereological estimation of the total number of neurons in thesubdivisions of the rat hippocampus using the optical fractionator. *Anat Rec* **231**, 482-497 (1991).

2. Ip CW, Cheong D & Volkmann J. Stereological Estimation of Dopaminergic Neuron Number in the Mouse Substantia Nigra Using the Optical Fractionator and Standard Microscopy Equipment. *J Vis Exp* (2017).

3. Ravier MA, Leduc M, Richard J, Linck N, Varrault A, Pirot N et al. beta-Arrestin2 plays a key role in the modulation of the pancreatic beta cell mass in mice. *Diabetologia* **57**, 532-541 (2014).

4. Leduc M, Richard J, Costes S, Muller D, Varrault A, Compan V et al. ERK1 is dispensable for mouse pancreatic beta cell function but is necessary for glucose-induced full activation of MSK1 and CREB. *Diabetologia* **60**, 1999-2010 (2017).

5. Kim D, Langmead B & Salzberg SL. HISAT: a fast spliced aligner with low memory requirements. *Nat Methods* **12**, 357-360 (2015).

6. Kim D, Paggi JM, Park C, Bennett C & Salzberg SL. Graph-based genome alignment and genotyping with HISAT2 and HISAT-genotype. *Nat Biotechnol* **37**, 907-915 (2019).

7. Liao Y, Smyth GK & Shi W. featureCounts: an efficient general purpose program for assigning sequence reads to genomic features. *Bioinformatics* **30**, 923-930 (2014).

8. Gentleman RC, Carey VJ, Bates DM, Bolstad B, Dettling M, Dudoit S et al. Bioconductor: open software development for computational biology and bioinformatics. *Genome Biol* **5**, R80 (2004).

9. Love MI, Huber W & Anders S. Moderated estimation of fold change and dispersion for RNA-seq data with DESeq2. *Genome Biol* **15**, 550 (2014).

10. Ge SX, Jung D & Yao R. ShinyGO: a graphical gene-set enrichment tool for animals and plants. *Bioinformatics* **36**, 2628-2629 (2020).
